# A critical evaluation of health risk assessment of modified mycotoxins with a special focus on zearalenone

**DOI:** 10.1007/s12550-018-0328-z

**Published:** 2018-09-13

**Authors:** Nicole Lorenz, Sven Dänicke, Lutz Edler, Christoph Gottschalk, Eva Lassek, Doris Marko, Michael Rychlik, Angela Mally

**Affiliations:** 1 German Federal Institute for Risk Assessment (BfR), Max-Dohrn-Str. 8-10, 10589 Berlin, Germany; 2 Institute of Animal Nutrition, Friedrich-Loeffler-Institute (FLI), Federal Research Institute for Animal Health, Bundesallee 50, 38116 Braunschweig, Germany; 3 Division of Biostatistics, German Cancer Research Center, Im Neuenheimer Feld 280, 69120 Heidelberg, Germany; 4 Chair of Food Safety, Veterinary Faculty, Ludwig-Maximilians-University Munich, Schönleutnerstr. 8, 85764 Oberschleissheim, Germany; 5 Bavarian Health and Food Safety Authority, Luitpoldstr. 1, 97082 Würzburg, Germany; 6 Department of Food Chemistry and Toxicology, University of Vienna, Währingerstr. 38, 1090 Vienna, Austria; 7 Chair of Analytical Food Chemistry, Technical University Munich, Alte Akademie 10, 85354 Freising, Germany; 8 Department of Toxicology, University of Würzburg, Versbacher Strasse 9, 97078 Würzburg, Germany

**Keywords:** Modified mycotoxins, Health risk assessment, Zearalenone, Biomonitoring, Combinatory effects

## Abstract

A comprehensive definition introducing the term “modified mycotoxins” to encompass all possible forms in which mycotoxins and their modifications can occur was recently proposed and has rapidly gained wide acceptance within the scientific community. It is becoming increasingly evident that exposure to such modified mycotoxins due to their presence in food and feed has the potential to pose a substantial additional risk to human and animal health. Zearalenone (ZEN) is a well-characterized *Fusarium* toxin. Considering the diversity of modified forms of ZEN occurring in food and feed, the toxicologically relevant endocrine activity of many of these metabolites, and the fact that modified forms add to a dietary exposure which approaches the tolerable daily intake by free ZEN alone, modified forms of ZEN present an ideal case study for critical evaluation of modified mycotoxins in food safety. Following a summary of recent scientific opinions of EFSA dealing with health risk assessment of ZEN alone or in combination with its modified forms, uncertainties and data gaps are highlighted. Issues essential for evaluation and prioritization of modified mycotoxins in health risk assessment are identified and discussed, including opportunities to improve exposure assessment using biomonitoring data. Further issues such as future consideration of combinatory effects of the parent toxin with its modified forms and also other compounds co-occurring in food and feed are addressed. With a particular focus on ZEN, the most pressing challenges associated with health risk assessment of modified mycotoxins are identified and recommendations for further research to fill data gaps and reduce uncertainties are made.

## Introduction

From the perspective of consumer health protection, it is essential that comprehensive assessment of human and animal health risks related to dietary intake of mycotoxins covers all forms of mycotoxins potentially causing adverse effects in humans and/or animals. In recent years, it has become increasingly evident that matrix-associated mycotoxins as well as chemical and biological modifications of the parent mycotoxin (e.g., thermal modifications during processing; fungus-, plant- or animal-derived metabolites)-collectively defined as “modified mycotoxins” (Rychlik et al. 2014)-may co-occur in addition to the corresponding “free” or parent compound and may contribute to overall mycotoxin exposure (EFSA 2014).

Thus, the presence of modified mycotoxins in food and feed has raised concern that modified mycotoxins may pose a non-negligible additional risk to human and animal health. Consequently, national and international agencies, including the European Food Safety Authority (EFSA) and the German Federal Institute for Risk Assessment (BfR), have launched efforts to address this emerging issue in food safety by developing strategies of how to evaluate potential added health risks due to the presence of modified mycotoxins, recognizing that poor availability of occurrence and toxicity data poses a specific shortcoming. For a critical assessment of modified mycotoxins in food safety, zearalenone (ZEN) and its modified forms are chosen as exemplary mycotoxins of key concern.

**Fig. 1:**
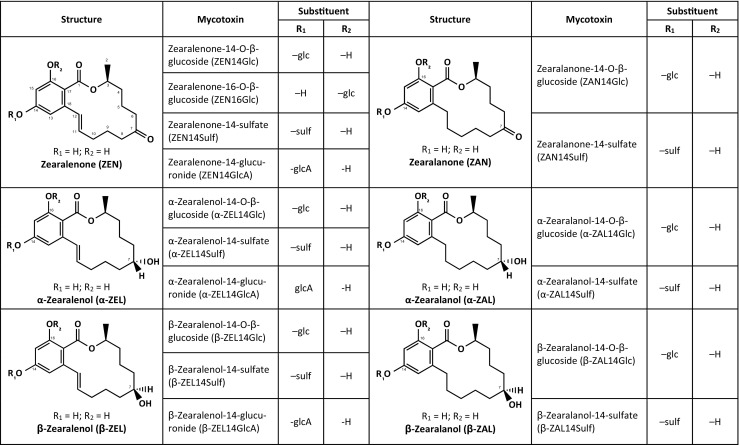
Overview on the chemical structures of zearalenone (ZEN) and its (currently known) modified forms (*glc* glucose, *sulf* sulfate, *glcA* glucuronic acid) (nomenclature and abbreviations according to Metzler 2010)

ZEN is a resorcyclic acid lactone mycotoxin (Fig. 1) primarily produced by several *Fusarium* species that grow on cereal host plants primarily in the field but to a minor extent also during poor grain storage conditions. Whereas ZEN exhibits low acute toxicity, long-term exposure to ZEN is considered to pose a health risk due to its potent estrogenic activity. Dietary exposure estimates suggest that current exposure levels to ZEN may be close to the tolerable daily intake (TDI) for ZEN established by EFSA as of 0.25 μg/kg body weight (bw) (EFSA 2011). Moreover, recent studies indicate that ZEN metabolites, which are produced by the fungus or host plants during fungal infection and are neither routinely screened for nor regulated by legislation, may also be present in a variety of cereal-based foodstuffs or may be formed during food processing (Brodehl et al. 2014). These modifications include the reductive phase I metabolites α- and β-zearalenol (α- and β-ZEL), zearalanone (ZAN) and α- and β-zearalanol (α- and β-ZAL), and phase II metabolites such as glucosides and sulfates (Berthiller et al. 2006; EFSA 2016; Engelhardt et al. 1988; Plasencia and Mirocha 1991) (Fig. 1, Table 4). In mammalian organisms, conjugation of ZEN and its phase I metabolites with glucuronic acid presents a major route of biotransformation and hence ZEN-derived glucuronides may be present in animal-derived foods (EFSA 2017a) (Fig.1, Table 4). To date, more than 30 modified forms of ZEN are described (EFSA 2017a) including the correspondent *cis*-forms originating from *cis*-isomerization of the parent compound due to sunlight exposure (Drzymala et al. 2015). Importantly, modified forms of ZEN may also possess estrogenic potential, which could even exceed that of ZEN (as shown, e.g., for α-ZEL; Table 4). These findings further underscore the potential contribution of modified forms of ZEN to health risks related to the presence of these mycotoxins in food and feed.

Considering the range of modified forms of ZEN detected in food and feed, the endocrine activity of several of these metabolites and the fact that estimated dietary exposure to free ZEN alone is already close to the TDI, ZEN was identified as an exemplary mycotoxin of key concern to present a critical evaluation of modified mycotoxins in food safety. Following a summary of recent scientific opinions published by EFSA dealing with health risk assessment of ZEN alone or in combination with its modified forms, uncertainties and data gaps are highlighted, and issues essential for the evaluation and prioritization of modified mycotoxins in health risk assessment are identified and discussed. Since major uncertainties in the current health risk assessment of ZEN and its modified forms stem from limited occurrence and exposure data, biomonitoring is considered as a supportive approach to overcome methodological limitations and uncertainties in the current exposure assessment. To this end, an overview of currently available biomonitoring data on ZEN and its modified forms in humans and farm animals is presented and opportunities and shortcomings in using biomonitoring data for exposure assessment of ZEN and its modified forms are discussed. A further, currently neglected aspect of health risk assessment associated with dietary exposure to modified mycotoxins relates to potential combinatory effects with other contaminants and undesirable substances in food. This appears particularly important for ZEN and its modified forms since humans are exposed to a wide variety of chemicals that are known to interfere with the endocrine system, including phytoestrogens that sometimes co-occur with ZEN in food. Although information on combinatory toxic effects and co-occurrence of ZEN and its modified forms with other xenoestrogens in food is still scarce, all currently available data are summarized and discussed. Finally, key challenges are highlighted and recommendations for further research required to reliably assess the additional health risk related to modified mycotoxins, especially of modified forms of ZEN, are given.

## Recent scientific opinions of EFSA on ZEN and its modified forms

Human health risk from ZEN in food was re-assessed by EFSA in 2011 in a follow up of previous assessments of the Scientific Committee on Food (SCF 2000) and the 53rd Meeting of the Joint Food and Agriculture Organization of the United Nations/World Health Organization Expert Committee on Food Additives (WHO 2000). Based on comprehensive hazard identification and characterization, the estrogenic activity of ZEN was identified as the critical mode of action of ZEN (and its main reductive metabolites) responsible for the endocrine, reproductive, and developmental toxicity of ZEN observed in experimental and farm animals (EFSA 2011).

Female pigs were identified as most sensitive to the estrogenic effects of ZEN in their ovaries, uterus, and vulva, and any estrogenic disturbance was considered at that time as the critical effect for the health risk assessment of humans related to the presence of ZEN in food. The study of Döll et al. (2003) on female piglets exposed to ZEN at doses of 0.5, 3.0, 7.4, 10.4, and 17.6 μg ZEN/kg bw per day from naturally contaminated maize for 5 weeks was chosen as the pivotal study to determine a reference point for human health risk characterization for ZEN. Considering the number of piglets with swollen and reddened vulva and cervix and the uterus weights (both on an absolute and relative bw basis), the second highest dose of 10.4 μg ZEN/kg bw per day was identified as a no-observed-effect-level (NOEL) in this study. A NOEL was identified rather than a no-observed-adverse-effect-level (NOAEL) since the observed estrogenic effects were not considered adverse in terms of later fertility and reproductive performance. However, the estrogenic effects were considered as undesirable and indicative of adverse effects and thus appropriate for human hazard characterization and derivation of a TDI for chronic exposure to ZEN. Using 10 μg ZEN/kg bw per day as a point of departure, an uncertainty factor of 4 for toxicokinetic differences between pigs and humans (interspecies extrapolation) and an uncertainty factor of 10 for interhuman variability, EFSA established a TDI of 0.25 μg/kg bw per day in 2011. The approach taken by EFSA did not adjust for toxicodynamic differences between pigs and humans since endogenous 17β-oestradiol plasma concentrations during the menstrual cycle in humans appear to be within the same order of magnitude as during the estrous cycle in pigs and hence it was considered unlikely that women are more sensitive to estrogens than female pigs. Furthermore, co-exposure to deoxynivalenol (DON) present in the naturally contaminated feed (Döll et al. 2003) was regarded as unlikely to influence the value of the NOEL for ZEN as no effect on body weight was observed at this dose level. The significant reduction of body weight recorded at the highest dose was related to DON-mediated reduction in feed intake rather than effects of ZEN on the uteri.

Using the occurrence data available at that time, EFSA estimated the mean and 95th percentile chronic total dietary exposure to ZEN to range up to a maximum upper bound of 0.10 and 0.28 μg/kg bw per day, respectively, in toddlers representing the age group with the highest exposure estimates in humans. EFSA concluded that chronic dietary exposure to ZEN was below or at maximum in the region of the TDI and would thus not raise a health concern.

Significant sources of uncertainty in the current risk assessment of ZEN alone (i.e., not in combination with its modified forms) include the low proportion of quantified analytical results (only 15% of the occurrence data), data gaps in the occurrence data (especially for some relevant food groups) as well as an insufficient consideration of special consumer groups (especially vegetarians) leading to several recommendations for further research (Table 1).

**Table 1:** Overview on recommendations given by EFSA for further research concerning ZEN and its modified forms (EFSA 2011; EFSA 2014; EFSA 2016)

Reference	Recommendation
EFSA 2011	➢ Collection of more occurrence data on ZEN in soy and soy-based foods ➢ Obtainment of more food consumption data for vegetarians
EFSA 2014	There is a need for ➢ more information on the chemical structures of modified forms ➢ further work to identify modified mycotoxins not yet characterized ➢ standardized nomenclature including abbreviations for mycotoxins and their modified forms ➢ properly validated and sensitive routine analytical methods for modified mycotoxins ➢ investigation of the fate of modified mycotoxins upon food and feed processing ➢ more occurrence data on mycotoxins and their modified forms in food and feed ➢ toxicological data on modified mycotoxins
EFSA 2016	There is a need for ➢ more data on the occurrence of modified forms of ZEN in food (including food of animal origin) and feed in order to characterize risks using the group-TDI and the RPFs established in this opinion ➢ more data on toxicokinetics of modified forms of ZEN, particularly on the absorption and bioavailability of phase II metabolites of ZEN present in food and feed ➢ investigation of estrogenicity of modified ZEN, in particular α-ZEL, comparative to ZEN, in pigs, the most sensitive species for ZEN toxicity, to reduce the uncertainty associated with the establishment of the RPFs ➢ sufficiently sensitive analytical methods for the analysis given the high RPFs for some modified forms

Since 2011, it has become increasingly more evident that modified mycotoxins could also be present in food (as well as in feed) and may thus contribute to the overall toxicity of the respective parent compound. In 2014, EFSA for the first time evaluated the risks for human and animal health related to the presence of modified forms of certain Fusarium toxins, including ZEN and its modified forms, in food and feed (EFSA 2014). In the absence of toxicity data and based on the assumption that modified mycotoxins may be metabolized or released from the matrix during digestion (Dall’Erta et al. 2013; Gareis et al. 1990; Veršilovskis et al. 2012) and subsequently absorbed in the gastrointestinal tract primarily in form of the parent compound, EFSA considered it appropriate to assume that modified mycotoxins exhibit the same toxicity as their parent compounds.

EFSA estimated chronic dietary exposure to ZEN and its modified forms by adding 100% to the previous exposure estimates for ZEN (EFSA 2011) to account for the presence of modified ZEN in food. This addition of 100% was based on only very few literature data on the co-occurrence of modified forms of ZEN (in particular ZEN14Glc, ZEN14Sulf, α-ZEL, α-ZELGlc, β-ZEL, and β-ZELGlc) from which a total concentration of up to 110% of the parent compound was calculated (De Boevre et al. 2012, 2013; Schneweis et al. 2002; Streit et al. 2013; Vendl et al. 2010). Much lower additions of 10%, 30%, and 60% were added to account for the modified forms of the other Fusarium toxins T2/HT2, nivalenol, and fumonisins, respectively.

Using the lower bound (LB) approach, mean and 95th percentiles exposure estimates of ZEN together with its modified forms were still below the TDI for ZEN in all age groups. However, at the maximum upper bound (UB), the estimates of high exposure at the 95th percentile exceeded the TDI of 0.25 μg/kg bw by a factor of 1.7 and 2.2 in infants and toddlers, respectively. Thus, a possible health concern for special consumer groups could not be excluded.

EFSA concluded that modified forms of ZEN may substantially contribute to the overall exposure, but emphasized also that the overall uncertainty was high. Therefore, the evaluation was considered as a “pragmatic approach” to provide a first assessment of the health risk related to modified mycotoxins present in food. EFSA (2014) identified several uncertainties (e.g., limited data from the literature on the occurrence of modified mycotoxins in food or equal toxicity of all modified forms compared to ZEN) resulting in numerous recommendations for further research before including modified mycotoxins in risk characterization (Table 1).

In 2016, EFSA assessed whether it is appropriate to set a group health-based guidance value (in this case a group-TDI) for ZEN and its modified forms and to use ZEN as a marker for the presence and the extent of toxicity of ZEN and its modified forms (EFSA 2016). Following a systematic review of relevant data on ZEN published after the 2011 assessment, it was concluded that new studies did not provide evidence for a need to revise the current TDI for ZEN of 0.25 μg/kg bw per day.

In light of very limited data on the absorption, bioavailability, and metabolism of modified forms of ZEN in animals or humans, EFSA assumed that phase I metabolites of ZEN are as bioavailable as ZEN, and that conjugated forms of ZEN become equally bioavailable as their unconjugated forms following hydrolysis in the gastrointestinal tract. The phase I metabolites were ranked for their oestrogenic potential as follows: α-ZEL > α-ZAL ≈ *cis*-α-ZEL > ZEN ≈ ZAN ≈ *cis*-ZEN ≈ β-ZAL ≈ *cis*-β-ZEL > β-ZEL. Phase II metabolites, which may acquire estrogenic activity after cleavage in the gastrointestinal tract to ZEN or its phase I metabolites, were regarded as equally estrogenic as the respective unconjugated forms. In comparison to 17β-oestradiol, α-ZEL and ZEN are approx. 10 times and 600 times less estrogenic, respectively (Everett et al. 1987).

EFSA assumed that any combinatory effects of unconjugated ZEN metabolites would be additive and thus considered it “appropriate to set a group-TDI of 0.25 μg/kg bw per day expressed as ZEN equivalents for ZEN and its modified forms” (EFSA 2016). Each modified form was assigned a relative potency factor (RPF), derived from results of the uterotrophic assay in rats, to be applied to exposure estimates of the respective modified forms to reflect the different in vivo estrogenic potencies. ZEN as reference or index mycotoxin was assigned a RPF of 1.0, whereas a RPF of 60 was assigned to α-ZEL to account for its much higher estrogenic potency (Table 4). Phase II metabolites were assigned the same RPF as the corresponding unconjugated forms (Table 4).

As major sources of uncertainties which were reflected in the recommendations for further research (Table 1), EFSA (2016) identified the assumptions that hydrolysis of ZEN conjugates in the gastrointestinal tract is complete, that the oestrogenic activities of ZEN and its phase I metabolites are additive, and that the RPFs were derived from rats rather than pigs (the most sensitive animal species).

In 2017, EFSA for the first time applied the RPFs for assessing the health risk of farm and companion animals exposed to ZEN and its modified forms by calculating an RPF-weighted sum to characterize the combined risk of ZEN and its modified forms. As far as available, exposure estimates were then compared with NOAEL or LOAEL values derived from toxicity data available for farm and companion animals (EFSA 2017a).

## Current status of biomonitoring of ZEN and its modified forms in humans and farm animals

At present, realistic estimates of human dietary exposure to ZEN and its modified forms based on food consumption and occurrence data are hampered by a lack of commercially available analytical standards and certified reference materials and the absence of validated analytical methods for the simultaneous quantification of ZEN and its modified forms. Consequently, data on co-occurrence of ZEN and its modified forms are scarce and restricted to selected food items, which are not necessarily representative of all foods potentially contaminated by ZEN. For instance, there is some evidence that vegetarians may have higher exposures to ZEN (according to EFSA (2011) up to Twofold higher) and likely also to its modified forms. However, the present database on the occurrence of ZEN and its modified forms and on the consumption of respective food items by vegetarians (e.g., soy and soy-based foods) is rather poor. Importantly, special consumer groups with high intake of maize products were not considered in the assessments by EFSA. Children suffering from celiac disease may have an increased intake of maize products compared to the average population when substituting wheat products, likely resulting in higher exposure to ZEN and its modified forms.

In light of the methodical limitations and uncertainties in the current exposure assessment, consideration should be given to supportive approaches that do not rely on occurrence data and food consumption pattern alone. Biomonitoring of selected metabolites in body fluids as quantitative indicators of exposure appears particularly attractive to simultaneously assess overall exposure to both the parent mycotoxin and its modified forms independent of the source, origin, or entry path. This approach may also allow identification of vulnerable and highly exposed consumer groups and assessment of regional and temporal variability in human and animal exposure. Furthermore, biomonitoring offers the opportunity to determine internal mycotoxin exposure (i.e., the amount of a substance that becomes systemically available and may thus reach the target site of toxicity) rather than external exposure (i.e., the amount of a mycotoxin ingested orally). Considering the lack of data on oral bioavailability and toxicokinetics of modified forms of ZEN and the uncertainties arising from the assumption that conjugated forms are cleaved to 100% and become equally bioavailable as ZEN, this may present a significant advantage over the current approach to estimate exposure to ZEN and its modified forms. Thus, biomonitoring of ZEN and its modified forms may provide valuable information on human and animal exposure that complements exposure estimates based on occurrence and food consumption data.

### Biomonitoring of ZEN and its modified forms in humans

Biomonitoring relies on accurate measurement of a biomarker or a combination of biomarkers that correlate with external exposure. This requires both comprehensive understanding of the toxicokinetics of the compound(s) and sufficiently sensitive and validated analytical methods to determine the levels of the biomarkers (e.g., the parent compound and/or a metabolite) in a suitable matrix. Limited data on ZEN in humans derived from a single volunteer suggest that ZEN and its phase I metabolites α-ZEL and β-ZEL are excreted in urine mainly in the form of their glucuronides (Mirocha et al. 1981). Based on these data, it has been estimated that 10–20% of an oral dose of ZEN are excreted within 24 h (Metzler et al. 2010), indicating that urinary metabolites of ZEN may present suitable biomarkers to monitor human exposure to ZEN and possibly also to its modified forms. However, there are so far no data on toxicokinetics of modified forms of ZEN in humans.

Several studies have employed sensitive LC-MS/MS methods to quantify ZEN and its (human) metabolites in urine collected from different cohorts from various countries around the world. These include “dilute and shoot” approaches, which directly measure urinary ZEN excretion using analytical standards for all major urinary metabolites, and indirect approaches employing enzymatic deconjugation and enrichment of analytes prior to analysis of unconjugated ZEN and its metabolites. Direct methods typically established as multi-mycotoxin methods to monitor human exposure to a wide range of mycotoxins and their metabolites are generally less sensitive than indirect methods that are optimized for ZEN and its phase I metabolites α-ZEL and β-ZEL involving enrichment of analytes (until 2016 reviewed in Binder et al. 2017; Mally et al. 2016; Yan et al. 2018). The percentage of positive samples in human biomonitoring (HBM) studies, therefore, depends to a large extent on the analytical method employed. A comparative analysis of urines of a South African cohort highlighted marked differences in the percentage of positive samples using direct vs. indirect approaches (2 vs. 98% of samples tested positive, respectively) due to the higher sensitivity of the indirect analytical method (Shephard et al. 2013). Consequently, direct approaches may well detect ZEN and its metabolites in highly exposed individuals, but appear to be less suitable to monitor mean exposures to ZEN and its modified forms.

Indirect approaches of monitoring urinary ZEN, α-ZEL, and β-ZEL indicate the existence of geographical differences in mean levels of total urinary ZEN. Consistent with the higher exposure to ZEN in African countries due to the consumption of maize as a major staple food and climate conditions that favor fungal infection, significantly higher levels of urinary ZEN and its metabolites were observed in a South African cohort as compared to European cohorts (e.g., for ZEN: 0.204 ± 0.456 ng/ml in South Africa vs. 0.057 ± 0.023 ng/ml in Southern Italy) (Föllmann et al. 2016; Shephard et al. 2013; Solfrizzo et al. 2014; Wallin et al. 2015). Within Europe, slightly higher occurrence rates and levels of urinary ZEN were observed in individuals from Italy (0.057 ± 0.023 ng/ml) as compared to cohorts from Germany (0.031 ± 0.023 ng/ml) and Sweden (0.03 ± 0.06 ng/ml) (Föllmann et al. 2016; Solfrizzo et al. 2014; Wallin et al. 2015). Interestingly, exposure estimates based on ZEN occurrence and food consumption data also indicate a somewhat lower level of exposure in Scandinavian countries and Germany as compared to Italy (EFSA 2011).

Translation of urinary biomarker concentrations into human exposure estimates requires reliable human data on urinary excretion rates, which are at present not available for ZEN and its modified forms. Preliminary approaches to calculate probable daily intakes based on urinary ZEN concentrations suggest that some individuals in South Africa or Haiti may be exposed to ZEN at levels exceeding the TDI of 0.25 μg/kg bw, whereas ZEN exposure in European countries appears to be below the TDI (Gerding et al. 2015; Shephard et al. 2013; Solfrizzo et al. 2014). However, further validation of urinary metabolites of ZEN and its modified forms is needed before urinary biomarkers can be used to estimate oral exposure. In particular, there is a need for data on urinary excretion rates of ZEN and its metabolites in humans, including potential age- and gender-related differences.

In light of recent data indicating the presence of a range of ZEN modifications in food, including significant amounts of conjugates of ZEN and α- and β-ZEL (EFSA 2014) which may be cleaved to the non-conjugated compounds, it is evident that measuring ZEN alone may not adequately cover exposure from modified forms of ZEN. Based on the methods and data presently available, it appears that measuring urinary ZEN and α- and β-ZEL following enzymatic cleavage of their conjugates may provide a reasonable and comprehensive biomarker approach to monitor human exposure to ZEN and its modifications.

### Biomonitoring of ZEN and its modified forms in farm animals

In contrast to biomonitoring in humans for which individual oral exposure is generally unknown, experiments in farm animals offer the opportunity to directly relate oral exposure to ZEN and its modified forms to the corresponding mycotoxin residues in various matrices such as blood, urine, bile, feces, milk, and eggs. Particularly if designed as dose-response studies, biomonitoring data combined with oral exposure data enable the generation of regression equations (reviewed in Dänicke and Winkler 2015; Gambacorta et al. 2013) which can then be used to predict oral exposure based on the analysis of an appropriate biomarker in a physiological sample (bile, urine, plasma, or serum). This approach is well established for pigs (Brezina et al. 2014, 2016; Dänicke et al. 2005) and cows (Winkler et al. 2014a, b, 2015) and could be used by veterinary practitioners for diagnosis.

Which matrix and biomarker is most suited to estimate oral intake ultimately depends on the toxicokinetics of ZEN and its modified forms in the species of interest. While systemic blood would be the substrate of choice as residues found in this matrix closely reflect internal exposure at target tissues, the systemic blood concentration of ZEN, its modified forms, and metabolites thereof is relatively low, requiring sensitive analytical techniques. In contrast, enterohepatic cycling of ZEN metabolites leads to enrichment of ZEN and its metabolites in bile, making this matrix interesting for diagnostic purposes, despite the fact that collecting bile on a routine basis is difficult. While urine lends itself to monitoring approaches as it can be collected non-invasively, studies in animals show that the relationship between urinary residue concentrations and dietary exposure is more variable than that of bile (for further details, see Dänicke and Winkler 2015, EFSA 2017a, or Gambacorta et al. 2013).

Importantly, biomonitoring in farm animals revealed significant differences in toxicokinetics and metabolism between species, age groups, as well as genders within a species. In cattle, for instance, β-ZEL is the main phase I metabolite of ZEN (Mirocha et al. 1981; Winkler et al. 2015), whereas in pigs α-ZEL is the major reductive metabolite (Kollarczik et al. 1994; Mirocha et al. 1981). In human urine, comparable concentrations of α- and β-ZEL have been reported (Shephard et al. 2013; Solfrizzo et al. 2014; Wallin et al. 2015). Potential age-related differences in toxicokinetics and metabolism of ZEN and possibly also its modified forms are evident from studies in pigs. While piglets excrete mainly ZEN via urine (Brezina et al. 2016; Döll et al. 2003; Mirocha et al. 1981), the predominant urinary excretion product in mature gilts is α-ZEL (Dänicke et al. 2005; Goyarts et al. 2007). Finally, gender-specific or inter-individual variability in mycotoxin metabolism, including 3α-hydroxysteroid dehydrogenase-dependent biotransformation of ZEN (Malekinejad et al. 2006), may contribute to overall variability. This high variability coupled with limited number of samples analyzed so far hampers the transferability and generalization of currently available biomonitoring data along with extrapolation from (farm) animals to humans.

### Principal metabolic pathways of ZEN and its modified forms

**Fig. 2:**
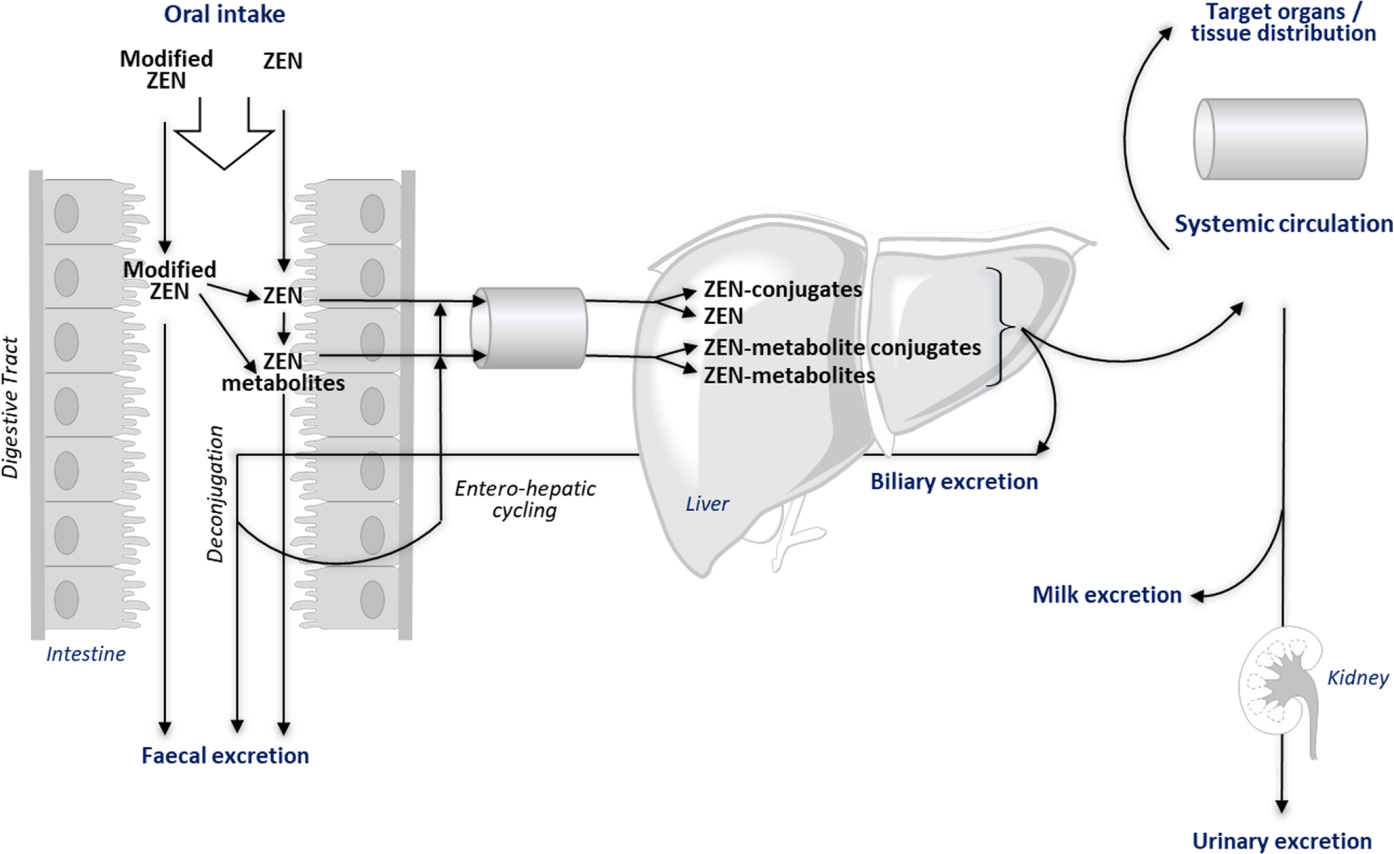
Principal metabolic pathways of ZEN and its modified forms (adopted from Dänicke and Winkler 2015)

As already pointed out, utilizing biomonitoring to obtain reliable exposure estimates requires sufficient knowledge of the toxicokinetics of the compound(s), including the identification of major routes of biotransformation and excretion (Fig. 2). While data on human metabolism and toxicokinetics of ZEN are already scarce, there is as yet even less information on the fate of modified forms of ZEN in humans following oral intake. Studies in animals not only demonstrate the complexity of metabolic conversion of ZEN and its modified forms (Fig. 2) but also highlight significant species differences. This renders extrapolation from animal to humans and estimation of total oral exposure to ZEN and its metabolites a challenging task.

Besides the intestinal mucosa and the liver, it is evident that gastrointestinal microbiota play an important role in the metabolism of ZEN and its modified forms (EFSA 2017a). Gastrointestinal metabolism primarily catalyzes formation of reductive metabolites and hydrolysis of glucosides and most likely also of sulfates, giving rise to the respective unconjugated forms. The degree of hydrolysis and reduction not only depends on species but is also subject to significant intra-species differences due to diet-associated variation in the composition and activity of gastrointestinal microbiota. Based on data obtained in animals and Caco-2 cells, it appears that ZEN and its reduced metabolites are rapidly and extensively absorbed from the gastrointestinal tract (Biehl et al. 1993; Pfeiffer et al. 2011). Further phase I metabolism via 3α- and 3β-hydroxysteroid dehydrogenases (HSDs) gives rise to reductive metabolites with a toxic potency that may even exceed the estrogenic potency of ZEN, whereas Cytochrome P450-mediated hydroxylation seems to present a minor metabolic pathway. Given the lower biological activity of ZEN glucuronides and sulfates compared to free ZEN (Frizzell et al. 2015; Plasencia and Mirocha 1991), uridine diphosphate-glucuronosyltransferase (UGT) and sulfotransferase (SULT)-dependent conjugation of ZEN and its reduced forms with glucuronic acid and sulfate may be seen as a mechanism of detoxification. However, endogenous cycling of estrogens between free and conjugated forms has been suggested as a putative activation mechanism at target cells (Thomas and Potter 2013; Zhu and Conney 1998), and it is possible that similar cycles of conjugation and hydrolysis of ZEN and ZEN-metabolite conjugates occur. Ultimately, the spectrum of metabolites in different target organs and body fluids is determined not only by the hepatic extraction rate but also by tissue-specific distribution of each individual metabolite. Consequently, both free and conjugated forms of ZEN and its metabolites need to be analyzed when considering biomonitoring data in health risk assessment.

The example of α-ZEL serves to illustrate the complexity of estimating oral exposure to ZEN and its modified forms based on biomarker data. In addition to being a modified form of ZEN to which humans and animals are exposed via food, α-ZEL is also a metabolite of ZEN and of various conjugated ZEN modifications such as ZEN14Glc, ZEN16Glc, ZEN14Sulf, α-ZEL14Glc, and α-ZEL14Sulf that co-occur in food and are deconjugated in the gut. Since current health risk assessment strategies rely on relative potency factors (RPFs) to be applied to exposure estimates of each respective modified form, it would be essential to reconstruct oral intake of each individual ZEN modification that would give rise to α-ZEL using reverse dosimetry. Clearly, this is a challenging task that requires comprehensive understanding of the absorption, distribution, metabolism, and excretion (ADME) of each modified form combined with sophisticated physiologically based pharmacokinetic (PBPK) modeling approaches.

## Current status of combinatory effects of ZEN and its modified forms with other (estrogenic) substances co-occurring in food

A critical but yet disregarded issue in health risk assessment of mycotoxins concerns combinatory effects not only of the parent mycotoxin with its modified forms but also with other substances present in food.

This appears particularly important in the case of ZEN and its modified forms since exposure to ZEN and its modified forms is estimated to be close to or even above the value of the group-TDI, at least for some age groups (EFSA 2011, 2014). Moreover, humans are exposed to a wide variety of chemicals that are known to interfere with the endocrine system, including other myco- and phytoestrogens that might co-occur with ZEN and is modified forms in food. Since endocrine disruption presents an important public health concern, this raises the critical question whether the current health risk assessment strategy, which focuses on ZEN and its modified forms only, is sufficiently protective for humans or whether more precise assessments of human health risks may be achieved by considering co-exposure to multiple oestrogenic substances, particularly during critical windows of exposure.

Co-occurrence of mycotoxins in food and feed is a well-known phenomenon (Alassane-Kpembi et al. 2017). However, due to the large number of known mycotoxins and the resulting complexity of the experimental designs of suitable studies for the investigation of combinatory effects (e.g., with respect to compound ratios, concentration ranges, selection of reasonable toxicological endpoints, etc.), comprehensive health risk assessment accounting for such co-occurrence remains a challenging task. To date, most data on combinatory effects of mycotoxins are from in vitro studies, and it remains to be seen whether the results can be confirmed in vivo.

With a primary focus on combinations of mycotoxins with comparably high prevalence but different modes of action (e.g., DON and ZEN), a vast number of different mycotoxin combinations have already been tested in vitro. The majority of these studies investigated primarily cytotoxic effects and/or related readouts in different cell models (e.g., Ren et al. 2017; Smith et al. 2017a, b, 2018; Wang et al. 2014; Zhang et al. 2018; Zhou et al. 2017). Based on these studies, it appears that the impact of the combination of DON and ZEN varies substantially depending on the ratio of test compounds applied, the concentration range, and the cell model, ranging from antagonistic to additive and even synergistic effects. In vivo studies on ZEN-containing mycotoxin mixtures focused primarily on potentially co-occurring *Fusarium* toxins (e.g., Gajecka et al. 2017; Liang et al. 2015; Szabo et al. 2018). Under in vivo conditions, the impact of ZEN together with other xenoestrogens remains largely unclear.

Importantly, ZEN not only co-occurs with other *Fusarium* toxins but, among others, also with non-regulated “emerging” mycotoxins such as alternariol (AOH), a mycotoxin formed by *Alternaria alternata.* In THP-1 cells, the combination of ZEN and DON with AOH was shown to inhibit differentiation from the monocyte status to macrophages, thus indicating immunomodulatory activity (Solhaug et al. 2016). Based on initial reports on estrogenic properties of AOH (Frizzell et al. 2013; Lehmann et al. 2006), a recent study investigated the combinatory effect of ZEN or α-ZEL with AOH in Ishikawa cells using the expression of alkaline phosphatase as a read-out for estrogenic activity (Vejdovszky et al. 2017a). In mixture with ZEN or α-ZEL, AOH was found to contribute to the overall oestrogenic activity over a broad concentration range (50 nM–10 μM). Depending on the concentration range and substance ratio, synergistic effects were also observed (e.g., 1 nM ZEN with all concentrations of AOH). The synergistic effect was even more pronounced in combinations of α-ZEL and AOH with strong synergism over a broad concentration range of α-ZEL (1 pM–1 nM). Only at very low concentrations, combinations of α-ZEL (10 pM) and AOH (2.5 nM) showed antagonistic effects. These data underline the complexity of potential interactions, which might substantially vary depending on concentration range and substance ratios. Notably, although ZEN, α-ZEL, and AOH as single compounds acted as partial agonist of the estrogen receptor, combinations of ZEN or α-ZEL with AOH reached or even exceeded the effect level of the full agonist 17β-oestradiol (1 nM) (Vejdovszky et al. 2017a).

Within the class of *Alternaria* mycotoxins, estrogenic properties seem not to be limited to AOH. A recent study shows that alternariol monomethyl ether, a known food contaminant, also acts as an oestrogenic stimulus in Ishikawa cells, even exceeding the oestrogenic activity of AOH (Vejdovszky et al. 2017a). These results are supported by in silico modeling, demonstrating respective fitting scores for binding to the estrogen receptor (Dellafiora et al. 2017). Furthermore, in silico modeling calculated binding scores for AOH-3-sulfate (AOH3Sulf) to the estrogen receptor alpha (ERα) that were in the range of AOH. AOH3Sulf represents a modified mycotoxin which might arise from fungal, plant, or mammalian metabolism (Dellafiora et al. 2017). Thus, the assessment of ZEN and its modified forms alone might underestimate the estrogenic potential of the fungal contamination of food or feed.

A further question, largely unexplored so far, is the impact of potentially co-occurring phytoestrogens, e.g., isoflavones in soy and soy-based products, 8-prenyl-naringenin in hops/beer, or secoisolariciresinol in linseed/breakfast cereals. A recent study investigated mycotoxin contamination in food supplements, reporting among others the occurrence of ZEN and AOH in a soy-based supplement (Veprikova et al. 2015). In a study on combinatory effects of the isoflavone genistein (GEN) and ZEN on the expression of the estrogen-sensitive alkaline phosphatase in Ishikawa cells showed substantial interactions of both compounds over a broad concentration range, whereby synergistic effects dominated (Vejdovszky et al. 2017b). A level of 10 nM GEN was found to be sufficient to significantly enhance the estrogenic stimulus mediated by ZEN (≥ 1 nM) to or even above the level of the natural ligand 17β-estradiol (1 nM).

These in vitro data demonstrate the influence but also the complexity of incorporating co-occurrence of xenoestrogens in the health risk assessment of ZEN and its modified forms.

## Evaluation of the current situation, identification of future challenges, and recommendations for further research

For consumer health protection, it is necessary to assess health risks of a substance as a priority if its potential toxicity is high (i.e., if it induces a critical toxicological effect or possesses high toxicological potential) and/or if potential human exposure is high (i.e., if its level in food is high or it occurs in frequently consumed food).

The example of ZEN demonstrates that modified mycotoxins may not only possess high toxicological potential (potentially even exceeding that of the parent compound) but may also significantly contribute to human and animal exposure. Thus, there is a clear need to assess the risks related to the co-occurrence of modified forms of mycotoxins. At present, however, data on the toxicity and occurrence of modified mycotoxins in food and feed are too limited to allow a scientifically sound conclusion. Nevertheless, exposure to modified mycotoxins is considered as an important emerging issue for consumer health protection. This is particularly true with regard to the estrogenic active mycotoxin ZEN und its modified forms for several reasons.

Using the upper bound (UB) approach, EFSA (2011) estimated that exposure to ZEN alone is (for some age groups) already in the region of the TDI established as a health-based guidance value for chronic dietary human exposure. Moreover, the value of the group-TDI, which was introduced to account for co-exposure to modified forms of ZEN, may even be exceeded by up to twofold (EFSA 2014). This appears especially critical as some relevant food groups, e.g., soy and soy-based products, were not included in the current exposure assessment due to a lack of appropriate occurrence data.

Importantly, the current exposure assessment of ZEN exhibits a high degree of uncertainty because only 15% of the occurrence data for ZEN in food utilized by EFSA in 2011 provided quantifiable results, whereas the other 85% were left-censored data. Given the relatively high values for the limit of detection (LOD) respectively the limit of quantification (LOQ), this leads to an unusual wide distance between the LB and UB exposure assessment with the consequence of overestimating exposure when utilizing the UB approach, only. For modified forms of ZEN, the number of food samples analyzed so far is yet much smaller and the results are even more diverse.

While exposure to the sum of modified forms of mycotoxins may be close to exposure to the parent mycotoxin (EFSA 2014), the different estrogenic potencies of the modified mycotoxins also need to be taken into account. This has been illustrated by the use of RPFs assigned to the different ZEN metabolites, which range from 0.2 for β-ZEL to 60 for α-ZEL, resulting in a factor of 300 (Table 4). With RPFs (much) higher than 1, some of the modified forms of ZEN may have an enormous impact on the overall exposure expressed as ZEN equivalents, even if their occurrence is low or moderate. Particularly when using the UB approach, in which the LOD/LOQ value is assigned to samples with undetectable levels, this could lead to a considerable overestimation of the actual exposure. Hence, the assumption of a possible health risk is largely a consequence of insufficient analytical sensitivity.

To overcome these shortcomings, six major challenges for reliable health risk assessment of modified forms of ZEN are identified and discussed in more detail below.

### Challenge 1: lack of standardized and sufficiently sensitive analytical methods

In 1990, Gareis et al. (1990) first determined ZEN14Glc in wheat by an indirect enzymatic approach. “Masked” ZEN was released after treatment with β-glucosidase and measured by HPLC-FLD in comparison with untreated samples. To date, mostly LC-MS/MS-based methods are used for the simultaneous direct determination of ZEN, its O-β-glucoside (ZEN14Glc) or sulfate (ZEN14Sulf), α-ZEL and β-ZEL, and their respective phase II metabolites in various cereal-based samples and in animal-derived food (Table 2). As an alternative approach, Beloglazova et al. (2013) proposed an immunoassay for the indirect determination of ZEN14Glc after enzymatic hydrolysis, which showed good correlation with results obtained by LC-MS/MS. Methods for other modified mycotoxins are not within the scope of this review, but were comprehensively reviewed in recent publications, e.g., of Berthiller et al. (2013).

**Table 2:** Overview on analytical methods for the determination of ZEN and its modified forms in food and feed (in chronological order)

Compounds^a^	Method of analysis–way of determination	Extraction	Clean-up	Sample matrices	Limit of detection	Reference
ZEN, ZEN14Glc, α-ZEL, β-ZEL	HPLC-FLD–direct/indirect (hydrolysis with b-glucosidase)	Chloroform	None	Cereals, feces, urine	nr	Gareis et al. 1990
ZEN, ZEN14Glc, β-ZEL	HPLC-FLD–direct	None	None	Analytical standard after chemical synthesis	nr.	Zill et al. 1990
ZEN, ZEN14Glc	LC-MS (ESI+)–direct	Acetonitrile/water 84/16 (v/v)	SPE (Florisil)	Wheat	10 μg/kg	Schneweis et al. 2002
ZEN, ZEN14Glc, ZEN14Sulf, α-ZEL, β-ZEL, α-ZEL14Glc, β-ZEL14Glc	LC-MS/MS (ESI−)–direct	Acetonitrile/water 75/25 (*v*/*v*)	None (direct measurement of an aliquot of the extract)	*Arabidopsis thaliana* (model plant)	nr	Berthiller et al. 2006
ZEN, ZEN14Glc, ZEN14Sulf, α-ZEL, β-ZEL	LC-MS/MS (ESI−)–direct	Acetonitrile/water/acetic acid 79/20/1 (*v*/*v*/*v*)	None (direct measurement of an aliquot of the extract)	Wheat, maize	0.03–2.0 μg/kg	Sulyok et al. 2006
ZEN, ZEN14Glc, ZEN14Sulf, α-ZEL, β-ZEL, α-ZEL14Glc, β-ZEL14Glc	LC-MS/MS (ESI−)–direct	Acetonitrile/water/acetic acid 79/20/1 (*v*/*v*/*v*)	None (direct measurement of an aliquot of the extract)	Pasta, popcorn, cornflour, porridge, beer	1.0–20 μg/kg	Vendl et al. 2009
ZEN, α-ZEL, β-ZEL	LC-MS/MS (ESI−)–direct	Acetonitrile/water 84/16 (*v*/*v*)	None (direct measurement of an aliquot of the extract)	Wheat, barley, spelt, oat, bread	2.0 μg/kg	Juan et al. 2012
ZEN, ZEN14Glc, ZEN14Sulf, α-ZEL, β-ZEL, α-ZEL14Glc, β-ZEL14Glc, ZAN	LC-MS/MS (ESI+)–direct	Acetonitrile/water/acetic acid 79/20/1 (*v*/*v*/*v*)	Defatting with hexane	Wheat, maize, oat, bread, cornflakes	5.0–13 μg/kg	De Boevre et al. 2012
ZEN, ZEN14Glc	ELISA–indirect (hydrolysis with glucosidase from *A. niger*)	Methanol/water 80/20 (v/v)	None (direct application of the extract after dilution with PBS)	Wheat, maize, breakfast cereals, feed	3.0 μg/kg	Beloglazova et al. 2013
ZEN, α-ZEL, β-ZEL, ZAN, α-ZAL, β-ZAL	LC-MS/MS (ESI−)–direct	Acetonitrile/water 90/10 (v/v)	SPE (BondElut Mycotoxin)	Muscle, liver, kidney, milk, eggs	0.2–0.4 μg/kg	Chen et al. 2013
ZEN, ZEN14Glc, ZEN14Sulf, α-ZEL, β-ZEL, α-ZEL14Glc, β-ZEL14Glc	LC-MS/MS (ESI−)–direct	Acetonitrile/water/acetic acid 79/20/1 (*v*/*v*/*v*)	None (direct measurement of an aliquot of the extract)	Cereal-based food and feed, silage	nr	Streit et al. 2013^b^
ZEN, α-ZEL	LC-MS/MS (ESI−)–direct	Acetonitrile (8 ml added to 2 g milk)	SPE (Oasis HLB)	Milk and milk powder	0.005 μg/kg	Huang et al. 2014

*nr* not reported

^a^Nomenclature of ZEN glucoside or sulfates adopted where appropriate

^b^The applied method was only validated for ZEN in apple puree for infants, hazelnuts, maize, green pepper (Malachová et al. 2014). Spectrum and performance criteria of metabolites were not explicitly listed

Apart from the aforementioned compounds, further metabolites of ZEN such as malonyl-glucosides or disaccharides were described in a plant model by Berthiller et al. (2006). Kovalsky Paris et al. (2014) recently reported ZEN16Glc as a new structure of ZEN-glucosides. In the applied plant cell model, ZEN16Glc levels were shown to be 18-fold higher than those of the previously known ZEN14Glc. This underlines the variability of metabolism of xenobiotics in plants as well as the necessity of ongoing analytical developments since studies on their occurrence in field samples are missing.

Method development is hampered by the limited availability of analytical standards and certified reference materials. Analytical standards for glucosides or sulfates of ZEN or glucosides of α-ZEL and β-ZEL are not commercially available. Chemical synthesis has been reported by Grabley et al. (1992), Mikula et al. (2013a, b, 2014), and Michlmayr et al. (2017). Alternatively, analytical standards can be produced by plant cell cultures (Engelhardt et al. 1988), by incubation of ZEN with yeasts (Poppenberger et al. 2006), or by cultivation of *Fusarium graminearum* on rice (Plasencia and Mirocha 1991). However, internal analytical standards designed for compensation of matrix effects in LC-MS/MS ionization are not available at all. Thus, LC-MS/MS analysis being subject to matrix interferences might be adversely influenced (Vendl et al. 2009). Periodically, ZAN was applied as internal analytical standard for covering analyte losses during clean-up as well as to compensate for matrix effects on ZEN and its metabolites (De Boevre et al. 2012). Whether this approach is suited for a due compensation of matrix effects should be considered with caution because of different chromatographic behavior of ZAN and ZEN-metabolites. Furthermore, there is a lack of knowledge on the stability of modified mycotoxins during storage and processing (Berthiller et al. 2013), which could also influence the evaluation of co-occurrence with their parent compounds. In this context, it is crucial to use aprotic solvents for storage of analytical standards since the conjugates are prone to hydrolysis to their parent compounds (Berthiller et al. 2013). Extraction of the conjugated mycotoxins usually requires more polar solvents than for extracting the parent compounds. Thus, a suited mixture of solvents is required, which provides satisfying and similar recoveries for all analytes. Otherwise, the comparison of results on the percentages of modified:parent compound might be misinterpreted. A mixture of acetonitrile/water/acetic acid 79/20/1 (*v*/*v*/*v*) is currently regarded the most suited (De Boevre et al. 2012; Vendl et al. 2009).

Vendl et al. (2009) tested various materials for sample clean-up. Due to the wide range of polarity of the compounds, none of the tested SPE-based methods proved suitable for the simultaneous determination. Immunoaffinity columns intended for the clean-up of ZEN showed acceptable cross-reactivities with α-ZEL and β-ZEL (> 55%), but were not suited for binding ZEN- or α/β-ZEL-glucosides or ZEN14Sulf (cross reactivity 0%). Results of Veršilovskis et al. (2011) were in line with the results of the aforementioned study after testing commercially available immunoaffinity columns. Thus, the majority of the multi-mycotoxin methods use dilute-and-shoot approaches instead of any clean-up procedure (Table 2).

### Recommendation 1: improvement of analytical methods

Analytical approaches for the direct or indirect determination of ZEN conjugates or metabolites are still very limited (Table 2), although a variety of modified forms of ZEN have already been identified. The development of standardized analytical methods and the availability of analytical standards and certified reference materials are crucial requirements for the joint quantification of ZEN and its modified forms per food sample as a reliable basis for realistic exposure assessment. Therefore, the development of validated and sufficiently sensitive analytical methods for the detection of modified mycotoxins in food, especially of modified forms of ZEN with an RPF ≥ 1, is highly recommended as the first and essential step for future research.

### Challenge 2: insufficient occurrence data for modified ZEN

The few comprehensive surveys of the co-occurrence of ZEN and its modifications in cereals and cereal-based foods originate mainly from De Boevre et al. (2012, 2013, 2014) and Vendl et al. (2010) (Table 3). These data were generated by in-house validated methods. Highest levels of contamination were found for maize with up to 15,700 μg/kg for free ZEN, 7970 μg/kg for the sum of α-ZEL and β-ZEL, and 9750 μg/kg for the sum of ZEN14Glc, ZEN14Sulf, α-ZEL14Glc, and β-ZEL14Glc (De Boevre et al. 2014). However, staple foods like bread and breakfast cereals were also shown to contain significant amounts of modified forms of ZEN along with free ZEN (Table 3). In some of the analyzed breads or breakfast cereals, the sum of modified forms of ZEN even exceeded the EU maximum limits for ZEN of 50 and 75 μg/kg, respectively (European Commission 2006).

**Table 3:** Overview on occurrence data on ZEN and its modified forms in various cereal-based matrices and animal products (all mean, median, and maximal concentrations are specified in μg/kg)

Sample (no of samples)	ZEN		α-ZEL		β-ZEL		ZEN14Glc		ZEN14Sulf		α-ZEL14Glc		β-ZEL14Glc		Reference
	Mean/Median	Max	Mean/Median	Max	Mean/Median	Max	Mean/Median	Max	Mean/Median	Max	Mean/Median	Max	Mean/Median	Max	
Cereals															
Barley (34)	13.7^a^	17	0.6^a^	0.6	2^a^	2	2.7^a^	9.6	11^a^	26	2.9^a^	5.1	0.7^a^	0.7	Nathanail et al. 2015
Maize (6)	270^b^	1071	63^b^	262	16.5^b^	103	nd^b^	274	nd^b^	51	nd^b^	283	55^b^	193	De Boevre et al. 2012
Maize (36)	925^b^	15,700	nq^b,c^	7970^c^			nq^b,d^	9750^d^							De Boevre et al. 2014
Maize (6)	nd^b^	6326	nd^b^	35	nd^b^	174	na	na	nd^b^	136	na	na	na	na	Streit et al. 2013
Oats (31)	77^a^	675	1.9^a^	2.3	3^a^	6	nq^a^	nq	32^a^	220	nd^a^	nd^a^	nd^a^	nd^a^	Nathanail et al. 2015
Oats (6)	29^b^	85	nd^b^	68	nd^b^	46	nd^b^	nd	nd^b^	12	nd^b^	nd	nd^b^	20	De Boevre et al. 2012
Wheat (30)	38^a^	234	0.6^a^	0.7	3.5^a^	6.9	0.6^a^	0.6	4.9^a^	23	3.1^a^	4.4	nd^a^	nd^a^	Nathanail et al. 2015
Wheat (6)	38^b^	109	nd^b^	16	nd^b^	49	nd^b^	nd	nd^b^	11	nd^b^	nd	nd^b^	nd	De Boevre et al. 2012
Wheat (24)	nr	860	na	na	na	na	nr	104	na	na	na	na	na	na	Schneweis et al. 2002
Cereal products															
Bran-enriched bread (36)	38^a^	157	6^a^	60	13^a^	96	18^a^	155	4^a^	143	0.3^a^	12	6^a^	153	De Boevre et al. 2013
Bran flakes (3)	40.1^b^	48.9	nd^b^	nd	nd^b^	nd	nd^b^	nd	5.8^b^	6.2	nd^b^	nd	nd^b^	nd	Vendl et al. 2010
Fiber-enriched bread (52)	29^a^	230	6^a^	110	7^a^	86	15^a^	154	4^a^	176	3^a^	63	7^a^	153	De Boevre et al. 2013
Bread (6)	31^b^	53	nd^b^	110	35^b^	96	nd^b^	20	nd^b^	24	nd^b^	nd	nd^b^	nd	De Boevre et al. 2012
Bread (10)	nd^b^	nq	nd^b^	nd	nd^b^	nd	nd^b^	nd	nq^b^	2.2	nd^b^	nd	nd^b^	nd	Vendl et al. 2010
Breakfast Cereals (62)	76^a^	450	43^a^	515	17^a^	147	39^a^	369	23^a^	417	11^a^	192	11^a^	206	De Boevre et al. 2013
Breakfast cereals (14)	nq^b^	44.2	nd^b^	nd	nd^b^	nd	nd^b^	nd	1.0^b^	6.1	nd^b^	nd	nd^b^	nd	Vendl et al. 2010
Cornflakes (6)	45^b^	90	nd^b^	34	47^b^	63	nd^b^	nd	nd^b^	nd	nd^b^	nd	nd^b^	nd	De Boevre et al. 2012
Crackers (3)	14.5^b^	16.0	nd^b^	nd	nd^b^	nd	nd^b^	nd	2.2^b^	2.9	nd^b^	nd	nd^b^	nd	Vendl et al. 2010
Oatmeal (13)	41^a^	85	10^a^	68	8^a^	46	12^a^	91	4^a^	36	1^a^	10	2^a^	10	De Boevre et al. 2013
Popcorn (12)	9^a^	51	3^a^	32	5^a^	47	nd^a^	nd	1^a^	12	nd^a^	nd	1^a^	10	De Boevre et al. 2013
Wheat flour (3)	nd^b^	18	nd^b^	nd	nd^b^	nd	nd^b^	nd	1.8^b^	3.5	nd^b^	nd	nd^b^	nd	Vendl et al. 2010
Wheat flour (3)	164^b^	200	na	na	na	na	na	na	45.9^b^	70.7	na	na	na	na	Schwake-Anduschus et al. 2015
Animal products															
Milk (42)	0.017^a^	0.046	0.028^a^	0.074	na	na	na	na	na	na	na	na	na	na	Huang et al. 2014
Milk powder (8)	0.012^a^	0.012	0.043^a^	0.064	na	na	na	na	na	na	na	na	na	na	Huang et al. 2014
Porcine muscle (10)	na	na	nd^b^	1.11	nd^b^	nd	na	na	na	na	na	na	na	na	Chen et al. 2013
Porcine liver (10)	na	na	nd^b^	2.23	nd^b^	nd	na	na	na	na	na	na	na	na	Chen et al. 2013
Milk (5)	na	na	nd^b^	nd	nd^b^	1.25	na	na	na	na	na	na	na	na	Chen et al. 2013
Eggs (5)	na	na	nd^b^	nd	nd^b^	2.51	na	na	na	na	na	na	na	na	Chen et al. 2013

*na* not analyzed, *nd* not detectable, *nq* not quantifiable, *nr* not reported

^a^Mean

^b^Median

^c^Sum of α-ZEL and β-ZEL

^d^Sum of ZEN14Glc, ZEN14Sulf, α-ZEL14Glc, β-ZEL14Glc

In comparison to α-ZEL and β-ZEL, their glucosides, i.e., α-ZEL14Glc and β-ZEL14Glc, showed a lower contribution to the overall exposure with percentages of less than 29% of ZEN (Table 3). Due to its relatively high LOQ values in LC-MS, only few research groups reported quantitative data for ZEN14Glc, which ranged between non-detectable (n.d.) and 51% relative to ZEN (Table 3). More data are available for ZEN14Sulf as it can be detected with superior sensitivity in LC-MS with contents amounting up to 30% of ZEN (Table 3). When calculating the sum of quantified levels of all modified forms with levels above LOD/LOQ, the amount of free ZEN could even be exceeded with levels up to 110% relative to that of ZEN (De Boevre et al. 2012, 2013).

Considering their high RPF of 60 (EFSA 2016), α-ZEL and α-ZEL14Glc are of particular concern. Their concentration was reported to be up to 71% of the ZEN content which indicates that these modifications may pose an even higher risk than ZEN itself.

For foods originating from animals, very few data are available. Interestingly, in milk and milk powder from China, α-ZEL has been reported to exceed the amount of ZEN by a factor of almost 4, although both contents were far below 1 μg/kg (Huang et al. 2014).

### Recommendation 2: generation of occurrence data

Overall, it can be concluded that only a very limited number of occurrence data on ZEN and its modified forms in food is published to date, which is mainly caused by a lack of standardized analytical methods. As a consequence of this shortcoming, modified mycotoxins are currently not integrated in monitoring programs or control measurements by official control laboratories for food and feed. There future inclusion is highly recommended to gain a broader database for comprehensive exposure assessment of ZEN and its modified forms.

A representative number of co-occurrence data (i.e., joint data on ZEN and its modifications in the same food sample), especially for food groups with high contamination levels or high consumption pattern, is urgently needed for a refined and more realistic exposure assessment. This may also help to identify possible marker substances for the co-occurrence of ZEN and its modified forms in food and feed.

Top priority should be given to food of plant origin, as recent studies suggest that the contribution of food of animal origin to the overall exposure is rather low (Dänicke and Winkler 2015). Accordingly, it is recommended to focus on obtaining occurrence data for biologically modified mycotoxins (especially modifications by plants and fungi).

### Challenge 3: toxicology of modified ZEN

In order to reliably assess the contribution of individual modified mycotoxins to health risks related to the presence of modified mycotoxins in food and feed, it is essential to understand both their toxicokinetics and toxic potential. Detailed understanding of the extent and form in which modified mycotoxins become bioavailable, as well as their biotransformation into active and non-active metabolites and route of excretion is equally important for hazard assessment and biomonitoring (see Challenge 5). Without consideration of oral bioavailability and metabolic conversion, in vitro studies may be of limited value for predicting comparative toxicity of modified mycotoxins in vivo. In addition, potential local effects of modified mycotoxins or their metabolites in the gastrointestinal tract may need to be considered.

Alternatively, substances with known structures but of unknown toxicity present in the diet at very low levels could be assessed with the threshold of toxicological concern (TTC) concept. However, based on current exposure estimates, it appears that the TTC concept is not suitable for the assessment of modified mycotoxins, emphasizing the need of substance-specific toxicity studies.

In light of the relatively large number of modified mycotoxins identified for ZEN, it does not appear realistic to assess the toxicokinetics and in vivo toxicity of all these modified forms. Rather, it seems reasonable to obtain high-quality in vivo data for a few exemplary modifications, including the most prevalent and the most potent forms (e.g., α-ZEL). This would enable the application of read-across, in silico prediction tools and in vitro bioactivity assays combined with physiologically based toxicokinetic (PBTK) modeling to estimate in vivo toxicity of the remaining modified forms.

With regard to modified forms of ZEN, it needs to be emphasized that derivation of the RPFs is so far based on results of the rat uterotrophic assay from a single study conducted in 1983. No data have been published on the in vivo toxicity of modified forms of ZEN in pigs, which were considered the most sensitive species for the oestrogenic effects of ZEN on which the TDI is based (see Challenge 4). Clearly, availability of sufficient amounts of analytical standards and certified reference materials is a key prerequisite for any in vivo or in vitro toxicokinetic and toxicity studies.

### Recommendation 3: investigation of toxicokinetics and in vivo estrogenicity

To reduce uncertainties in the toxicity of modified forms of ZEN, e.g., in the derivation of RPFs, more data on the estrogenicity and toxicokinetics of modified forms of ZEN are needed.

In vivo toxicokinetic studies investigating the oral bioavailability of (selected) conjugates are recommended to address uncertainties related to the assumption that ZEN conjugates become equally bioavailable as their unconjugated forms after hydrolysis in the gastrointestinal tract.

Considering the high oestrogenic potency of α-ZEL and thus potentially large contribution of α-ZEL to the overall health risk, comparative assessment of the oestrogenic effects of α-ZEL and ZEN in pigs is recommended.

### Challenge 4: derivation of appropriate health-based guidance values

Risk characterization, the final step of health risk assessment, integrates exposure estimates and health-based guidance values, e.g., a TDI. In 2016, EFSA extended the TDI for ZEN of 0.25 μg/kg bw to a group-TDI covering ZEN and its modified forms. EFSA considered derivation of the group-TDI as sufficiently conservative, because the estrogenic effects on which the TDI is based were considered to have no clear adverse consequences in terms of later fertility and reproductive performance and were seen as only “indicative” of an adverse effect (EFSA 2011). However, the study of Döll et al. (2003) identified as pivotal suffers from two major shortcomings that raise some concern about the appropriateness of this study as a basis for derivation of a health-based guidance value.

In this study, pigs were given feed naturally contaminated with ZEN. However, co-occurrence of modified forms of ZEN in the feed was not determined. Therefore, it cannot be excluded that effects of modified forms of ZEN potentially present in the diet were already (at least partially) included in the derivation of the TDI.

In addition to modified forms of ZEN, considerable amounts of other mycotoxins (e.g., deoxynivalenol, fumonisins, nivalenol) have been present in that naturally contaminated feed. The impact of these mycotoxins (and their modified forms) on the observed effects remains unclear. In 2011, EFSA stated that other mycotoxins would not be expected to have an impact because they possess no estrogenic potential. In 2016, however, EFSA added co-contamination with other *Fusarium* toxins to the list of uncertainties. Therefore, the impact of other *Fusarium* toxins should be scrutinized, especially in the light of the current discussion on combinatory effects.

### Recommendation 4: re-evaluation of the group-TDI

Considering the shortcomings of the pivotal feeding study in pigs, a re-evaluation of the value of the group-TDI is required, which accounts for the combined toxicities of ZEN and its modified forms.

New feeding studies in pigs are recommended which compare (at least) ZEN, α-ZEL, and the combination of ZEN and α-ZEL to be able to estimate whether the value of the group-TDI is appropriate for the health risk assessment of ZEN in combination with its modified forms.

### Challenge 5: utilization of biomonitoring data for exposure assessment

Considering the difficulties and uncertainties in the exposure assessment of mycotoxins based on occurrence in food and food consumption rates, there is increasing interest to utilize biomonitoring data as an efficient and cost-effective way to derive more reliable exposure estimates. Monitoring selected metabolites in body fluids as indicators of exposure appears particularly attractive to simultaneously assess overall exposure to both parent mycotoxins and their modified forms independent of the source, origin, or entry path; to identify vulnerable and highly exposed consumer groups; and to assess regional and temporal variability in human and animal exposure. At present, however, utilization of biomonitoring data to estimate exposure to modified forms of mycotoxins presents a major challenge.

Detection of relevant concentrations of ZEN metabolites in biological matrices at typical exposure levels requires very sensitive analytical methods, especially when applying analytical techniques which directly detect each modified form individually. Such approaches also require analytical standards for each modified form, and these are currently not commercially available (with the exception of α-ZEL and β-ZEL). While some of these shortcomings may be overcome by application of indirect methods which rely on enzymatic conversion of conjugated forms of ZEN into the respective phase I metabolites, indirect approaches will inevitably lead to a loss of information regarding the qualitative and quantitative composition of the mixture of modified mycotoxins present in the sample.

Moreover, validation of putative biomarkers of exposure for modified mycotoxins is complex, because the metabolite pattern detected in a particular physiological matrix (e.g., urine or blood) is the net result of the composition of the external exposure (pattern in food and feed) and the species-specific metabolism and toxicokinetics (Fig. 2). That is, some modified forms of ZEN such as α-ZEL occur both in feed and can also be generated in humans and animals from free ZEN. Furthermore, biomonitoring data of ZEN and its modified forms reveal not only differences in toxicokinetics and metabolism between species but also between age groups and genders within a species. Therefore, extrapolation from biomonitoring data achieved in animal experiments to humans is challenging and requires profound knowledge of intra- and interspecies-specific differences in metabolism and toxicokinetics of these mycotoxins. This further complicates validation of appropriate biomarkers of exposure. Thus, at present exposure assessment of mycotoxins, respectively ZEN and its modified forms, based on biomonitoring data alone would exhibit a high degree of uncertainty.

Due to the fact that the metabolite pattern of internal exposure (i.e., concentration and pattern of ZEN and its metabolites in systemic blood or urine) does not necessarily mirror the pattern of external exposure, derivation of toxicological reference values for the health assessment of the internal exposure calculated from biomonitoring data would be appreciated. To our knowledge, at present, no such “biomonitoring values” exist for mycotoxins. Therefore, internal exposure needs to be “translated” into external exposure using physiologically based toxicokinetic (PBTK) modeling approaches to allow a comparison with health-based guidance values, e.g., the TDI, which are derived from toxicological studies correlating a specific effect with a defined intake level.

Despite their current limitations, biomonitoring data could be seen as a valuable addition to the current health risk assessment practice that relies on external exposure estimates based on occurrence in food and consumption data. To the best of our knowledge for the first time in health risk assessment of mycotoxins, EFSA recently utilized human biomonitoring data as “supporting information” for the exposure assessment of deoxynivalenol (DON), its acetylated forms, and DON-3-glucoside (EFSA 2017b). In this opinion, EFSA applied DON-biomarker data to support the group-ARfD (acute reference dose) for DON and its modified forms derived from human acute outbreak data.

Moreover, biomonitoring would be expected to deliver additional data and important knowledge to close existing data gaps. To achieve this goal, systematic studies, preferably in humans, are needed that clearly link external and internal exposure. At present, however, such studies are scarce. For ZEN, only two studies, each consisting of a single male proband, have been reported (Mirocha et al. 1981; Warth et al. 2013). No data are available from female volunteers, even though significant gender-specific differences may be expected—especially in the case of the oestrogenic mycotoxin ZEN.

Finally, biomonitoring data might be used to reduce uncertainties in the current exposure assessment, particularly with regard to special consumer groups like vegetarians or patients suffering from coeliac disease. Conversely, the interpretation of biomonitoring data could be improved by using health risk assessment and in particular external exposure assessment, e.g., when elucidating entry pathways for mycotoxins or developing minimization strategies for the reduction of mycotoxin exposure.

### Recommendation 5: identification of reliable biomarkers of exposure and generation of representative biomonitoring data

The presently available biomonitoring data are too limited to close existing data gaps in health risk assessment but may help to reduce some uncertainties, e.g., concerning the exposure estimation of special consumer groups like vegetarians.

Generation of representative biomonitoring data and systematic studies (preferentially in humans and for ZEN and its modified forms also in pigs) are highly recommended to improve prediction of external and internal exposure using reliable biomarkers of exposure.

Thus, biomonitoring data have the potential to foster a more comprehensive health risk assessment.

### Challenge 6: health risk assessment of chemical mixtures

Finally, it is increasingly being recognized that further advancement of health risk assessment strategies that are at present mainly based on evaluation of single compounds, is needed to enable assessment of chemical mixtures co-occurring in food. However, in view of health risk assessment, the focus of investigations of combinatory effects should be on reasonable chemical mixtures. In the case of ZEN, this particularly means chemical mixtures that may influence the estrogenicity of ZEN.

In light of the large number of possible combinations and the complexity of the experimental study design (selection of reasonable toxicological endpoints, cell lines (for in vitro studies) or animal models (for in vivo studies), concentration ranges, compound ratios, etc.), a tiered approach for testing of combinatory effects is recommended. Such a tiered approach would start with identification and investigation of the most relevant combination, initially neglecting combinations of second and third orders which could be taken into account at a later stage.

For ZEN, such a tiered approach in order of rising complexity may start with investigating combinatory effects of ZEN with its modified forms, e.g., α-ZEL before moving on to the next order to test ZEN (and its modified forms) in combination with other mycoestrogens, e.g., alternariol. At the third level, combinatory effects of ZEN (and its modified forms) with other estrogenic compounds present in food such as phytoestrogens, e.g., genistein, metalloestrogens, e.g., Cd and xenoestrogens, e.g., phthalates, should be investigated. The fourth level would involve analysis of ZEN (and its modified forms) with substances that lack estrogenic activity but frequently co-occur with ZEN, such as other (*Fusarium*) mycotoxins, e.g., deoxynivalenol, fungal secondary metabolites (except mycotoxins), and contaminants or undesired substances in food.

Whereas the first three orders involve estrogenic substances which may have a direct effect on the estrogenicity of ZEN (and its modified forms), the fourth order includes combinations with substances acting by a different mode of action but that may nevertheless have an indirect effect on the toxicity or disposition (ADME) of ZEN (and its modified forms).

In this context, it should be noted that recent activities and analytical advances in science provide for opportunities to measure or model exposure to a wide range of chemicals throughout an individual’s lifetime-collectively referred to as the exposome (NRC 2012; Wild 2005). Qualitative and quantitative understanding, of which individual compounds and chemical mixtures humans are exposed to, including consideration of different life-stages and life-styles, is expected to aid identification and prioritization of reasonable chemical mixtures for testing and subsequently for consideration in health risk assessment.

### Recommendation 6: identification and investigation of reasonable chemical mixtures

Investigation of reasonable chemical mixtures of ZEN (and its modified forms) with other (estrogenic) substances co-occurring in food and development of strategies for the health risk assessment (including concepts for advanced exposure assessment) of these chemical mixtures is recommended.

It is evident that the presence of modified forms of mycotoxins in food and feed may pose a non-negligible additional risk to human health and is therefore an important emerging issue in health risk assessment. Table 4 provides a summary of the current knowledge and illustrates shortcomings and existing data gaps for ZEN and its modified forms as mycotoxins of key concern. The most important and pressing challenge when assessing modified forms of ZEN is the development of sufficiently sensitive analytical methods for the detection of modified mycotoxins (1) to allow generation of reliable occurrence data for all relevant food groups (2). Further key challenges relate to reduction of uncertainties in the toxicity of modified mycotoxins (3) in order to derive appropriate health-based guidance values (4), utilization of biomonitoring data as a supporting approach to reduce uncertainties in the exposure assessment of modified mycotoxins (5), as well as development of strategies for health risk assessment of chemical mixtures (6).

**Table 4:** Overview on ZEN and its modified forms concerning their classification (according to Rychlik et al. 2014), relative potency factors (according to EFSA 2016), (commercial) availability as analytical standards, as well as occurrence in food and feed (according to references in Table 3)

Mycotoxin compound	Classification	Relative potency factor (RPF)^a,b^	Availability as analytical standard	Occurrence^c^
Zearalenone (ZEN)	Free mycotoxin	1.0	Yes	100%
ZEN-glucosides	Biologically modified; conjugated by plants	1.0	No	Up to 51%
ZEN-sulfates	Biologically modified; conjugated by plants, fungi, animals	1.0	No	Up to 30%
ZEN-glucuronides	Biologically modified; conjugated by animals	1.0	No	Not known
α-Zearalenol (α-ZEL)	Biologically modified; functionalized	60.0	Yes	Up to 51%
α-ZEL-glucosides	Biologically modified; conjugated by plants and fungi	60.0	No	Up to 29%
α-ZEL-sulfates	Biologically modified; conjugated by plants, fungi, animals	60.0	No	Not known
α-ZEL-glucuronides	Biologically modified; conjugated by animals	60.0	No	Not known
β-Zearalenol (β-ZEL)	Biologically modified; functionalized	0.2	Yes	Up to 40%
β-ZEL-glucosides	Biologically modified; conjugated by plants and fungi	0.2	No	Up to 29%
β-ZEL-sulfates	Biologically modified; conjugated by plants, fungi, animals	0.2	No	Not known
β-ZEL-glucuronides	Biologically modified; conjugated by animals	0.2	No	Not known
Zearalanone (ZAN)	Biologically modified; functionalized	1.5	Yes	Not known
ZAN-glucosides	Not known	1.5	No	Not known
ZAN-sulfates	Not known	1.5	No	Not known
α-Zearalanol (α-ZAL)	Biologically modified; functionalized	4.0	Yes	Not known
α-ZAL-glucosides	Not known	4.0	No	Not known
α-ZAL-sulfates	Not known	4.0	No	Not known
β-Zearalanol (β-ZAL)	Biologically modified; functionalized	2.0	Yes	Not known
β-ZAL-glucosides	Not known	2.0	No	Not known
β-ZAL-sulfates	Not known	2.0	No	Not known

^a^RPFs were expressed on a molar basis of the mycotoxins meaning, e.g., that 1 mol of α-ZEL is equivalent in its estrogenicity to 60 mol of ZEN, and 1 mol of β-ZEL is equivalent to 0.2 mol of ZEN and so on. Weight-related RPFs consequently mean that 1 g of α-ZEL is equivalent in its estrogenicity to 60 g of ZEN, and 1 g of β-ZEL is equivalent to 0.2 g of ZEN, respectively. Hence this RPFs show that particularly α-ZEL is 60 times more potent, while β-ZEL is 5 times less potent then ZEN

^b^In comparison to 17β-oestradiol, α-ZEL and ZEN are approx. 10 times and 600 times less oestrogenic, respectively (Everett et al. 1987)

^c^Occurrence is expressed as percentage of ZEN in various matrices

Several of these challenges and recommendations, such as the development of sufficiently sensitive analytical methods or the utilization of appropriate biomonitoring data as supportive information, are not limited to ZEN and its modified forms, but are also applicable for the health assessment of other modified mycotoxins. Other challenges and recommendations are more specific to ZEN due to the unusual high differences in the toxicological potential of ZEN and some of its modified forms, especially of α-ZEL. Depending on the outcome of the health risk assessments, risk management options have to be discussed in the future, e.g., the setting of additional maximum levels for the most potent modified mycotoxins or the derivation of consumption recommendations for special consumer groups.

## Abbreviations

ADME: Absorption distribution metabolism excretion
AOH: Alternariol
AOH3Sulf: Alternariol-3-sulfate
bw: Bodyweight
DON: Deoxynivalenol
EFSA: European Food Safety Authority
HBM: Human biomonitoring
GEN: Genistein
HSD: Hydroxysteroid dehydrogenase
LB: Lower bound
LO(A)EL: Lowest observed (adverse) effect level
NO(A)EL: No observed (adverse) effect level
PBTK: Physiologically based toxicokinetic
RPF: Relative potency factor
SULT: Sulfotransferase
TDI: Tolerable daily intake
UB: Upper bound
UGT: Uridine diphosphate-glucuronosyltransferase
ZEN: Zearalenone
ZEN14Glc: Zearalenone-14-O-β-glucoside
ZEN16Glc: Zearalenone-16-O-β-glucoside
ZEN14Sulf: Zearalenone-14-sulfate
ZEN14GlcA: Zearalenone-14-glucuronide
α-ZEL: α-Zearalenol
α-ZEL14Glc: α-Zearalenol-14-O-β-glucoside
α-ZEL14Sulf: α-Zearalenol-14-sulfate
α-ZEL14GlcA: α-Zearalenol-14-glucuronide
β-ZEL: β-Zearalenol
β-ZEL14Glc: β-Zearalenol-14-O-β-glucoside
β-ZEL14Sulf: β-Zearalenol-14-sulfate
β-ZEL14GlcA: β-Zearalenol-14-glucuronide
ZAN: Zearalanone
ZAN14Glc: Zearalanone-14-O-β-glucoside
ZAN14Sulf: Zearalanone-14-sulfate
α-ZAL: α-Zearalanol
α-ZAL14Glc: α-Zearalanol-14-O-β-glucoside
α-ZAL14Sulf: α-Zearalanol-14-sulfate
β-ZAL: β-Zearalanol
β-ZAL14Glc: β-Zearalanol-14-O-β-glucoside
β-ZAL14Sulf: β-Zearalanol-14-sulfate
